# Thalamic but Not Subthalamic Neuromodulation Simplifies Word Use in Spontaneous Language

**DOI:** 10.3389/fnhum.2021.656188

**Published:** 2021-05-20

**Authors:** Hannes Ole Tiedt, Felicitas Ehlen, Michelle Wyrobnik, Fabian Klostermann

**Affiliations:** ^1^Department of Neurology, Motor and Cognition Group, Charité—Universitätsmedizin Berlin, corporate member of Freie Universität Berlin and Humboldt-Universität zu Berlin, Berlin, Germany; ^2^Department of Psychiatry, Jüdisches Krankenhaus Berlin, Berlin, Germany; ^3^Institute of Psychology, Humboldt-Universität zu Berlin, Berlin, Germany; ^4^Berlin School of Mind and Brain, Humboldt-Universität zu Berlin, Berlin, Germany

**Keywords:** thalamus, language, deep brain stimulation, lexical frequency, frequency effect, lexical access, spontaneous language

## Abstract

Several investigations have shown language impairments following electrode implantation surgery for Deep Brain Stimulation (DBS) in movement disorders. The impact of the actual stimulation, however, differs between DBS targets with further deterioration in formal language tests induced by thalamic DBS in contrast to subtle improvement observed in subthalamic DBS. Here, we studied speech samples from interviews with participants treated with DBS of the thalamic ventral intermediate nucleus (VIM) for essential tremor (ET), or the subthalamic nucleus (STN) for Parkinson’s disease (PD), and healthy volunteers (each *n* = 13). We analyzed word frequency and the use of open and closed class words. Active DBS increased word frequency in case of VIM, but not STN stimulation. Further, relative to controls, both DBS groups produced fewer open class words. Whereas VIM DBS further decreased the proportion of open class words, it was increased by STN DBS. Thus, VIM DBS favors the use of relatively common words in spontaneous language, compatible with the idea of lexical simplification under thalamic stimulation. The absence or even partial reversal of these effects in patients receiving STN DBS is of interest with respect to biolinguistic concepts suggesting dichotomous thalamic vs. basal ganglia roles in language processing.

## Introduction

Deep brain stimulation (DBS) is an effective treatment option for disabling movement disorders, most frequently applied to the subthalamic nucleus (STN) and thalamic ventral intermediate nucleus (VIM) in patients with Parkinson’s disease (PD) and essential tremor (ET), respectively (Ashkan et al., 2017). Follow-up studies in patient groups with DBS applied to either one of these target structures have consistently demonstrated mild to moderate language impairment, in particular reduced verbal fluency (VF) output (e.g., Tröster et al., 1998; Ardouin et al., 1999; Fields et al., 2003; Woods et al., 2003; Funkiewiez et al., 2004; De Gaspari et al., 2006; Lefaucheur et al., 2012). Whereas these observations can to some extent be attributed to frontal brain lesions occurring during the surgical procedure (Okun et al., 2009; York et al., 2009; Witt et al., 2013; Le Goff et al., 2015), the effects of the stimulation itself appear to differ between thalamic and extra-thalamic DBS target sites. Thus, some investigations have reported subtle improvements of word naming or VF tasks associated with STN DBS in contrast to additional negative effects induced by VIM DBS (Mikos et al., 2011; Silveri et al., 2012; Ehlen et al., 2014). This differential impact of thalamic and subthalamic DBS is consistent with models defining language as an integrative function of a corticobasal network (Mesulam, 1990; Crosson, 1999; Parvizi, 2009; Poeppel et al., 2012) with dichotomous roles of the thalamus and the basal ganglia on the subcortical side.

In addition to the analysis of formal language testing procedures such as (and most commonly) VF tasks in the majority of available research, relatively few studies have investigated natural language. A neurolinguistic analysis of semi-structured interviews conducted in a VIM DBS group under active and inactivated VIM DBS revealed that DBS interfered with sentence formation, resulting in a simplified syntactic structure of language (Ehlen et al., 2016). With respect to STN DBS, the available studies have yielded inconsistent results, showing either no alterations, improvement, or deterioration of language under active stimulation (Zanini et al., 2003, 2009; Whelan et al., 2005; Phillips et al., 2012; Schulz et al., 2012; Batens et al., 2014, 2015). Whereas the larger part of the observed changes relates to grammar or morphosyntactic processing, linguistic parameters reflecting lexicality were mostly unaltered. Although it is therefore difficult to draw conclusions about the role of DBS for lexical or semantic performance, some studies hint at changed use of open and closed class words associated with both thalamic and subthalamic DBS. Open class words (e.g., nouns, full verbs, adjectives) are related to semantic information, whereas closed class words (e.g., conjunctions, articles, copula and modal verbs) mainly convey structural information and are limited with respect to the extent to which they can be varied in natural language (Garrett, 1990; Fanselow and Staudacher, 2008; Dürscheid, 2012). Apparently, the use of open class words was increased by STN DBS, but in contrast decreased in individuals with VIM DBS when being ON stimulation (Batens et al., 2014, 2015; Ehlen et al., 2016). Since the above study results were based on comparisons with normative data and did not reach statistical significance between ON and OFF conditions, the question of how DBS might interfere with lexical access remains open. Such an effect on lexico-semantic processes underlying word selection, however, is suggested by observations in patients with aphasic symptoms due to ischemic or hemorrhagic thalamic lesions which frequently include semantic paraphasia and naming deficits (Crosson et al., 1997; Raymer et al., 1997; Crosson, 1999); for a review see Crosson (2013). In the study of Raymer et al. (1997) two patients with thalamic aphasia performed normally during tasks requiring either lexical or semantic processing, but were markedly impaired in naming items with a low as compared to medium or high frequency of occurrence; this particular pattern was interpreted as indicating a deficit at a processing level for interfacing lexical and semantical information (Raymer et al., 1997), see also Crosson (2019). An investigation of lexical frequency, however, was not included in the above-mentioned studies of linguistic effects of (thalamic or subthalamic) DBS. The “frequency effect” defines facilitated retrieval or processing of words with higher as compared to low frequency of use—a robust effect that has consistently been demonstrated in a variety of experimental findings (e.g., Oldfield and Wingfield, 1965; Forster and Chambers, 1973; Jescheniak and Levelt, 1994; Morrison and Ellis, 1995). In view of its ubiquitous and consistent nature, usage frequency of words (i.e., lexical frequency) has been suggested to convey information about organizational principles of the “mental lexicon” (Allen et al., 1992) and lexical access in particular (Jescheniak and Levelt, 1994). Although different theoretical models have been proposed for language production, there is a relative consensus about distinct and sequential stages of this process: following the modular framework outlined by Levelt (1989), a “preverbal” message is formulated by accessing the mental lexicon to retrieve the appropriate words, which are finally structured into sentences. Word retrieval in particular is viewed as a two-stage process that consists of lemma selection and attaching the correct phonological structure (Garrett, 1975, 1990; Dell, 1986; Levelt, 1989). Localist connectionist models of language production have simulated this process as a spreading activation within a network of inter-connected units representing words in their meaning and sounds (e.g., Dell et al., 1997; Levelt et al., 1999; Foygel and Dell, 2000). Computational modeling of dysfunctional activation patterns underlying naming deficits or repetition errors in aphasia have supported a view of an interactive flow of information between the two stages of word production rather than discrete steps (Schwartz et al., 2006; Dell et al., 2007, for a review, see Schwartz, 2014). Lexical frequency effects may originate throughout this incremental process, beginning with the interface between the semantic (preverbal) and the lexical stage levels, with an emphasis, however, on phonological word form encoding (Jescheniak and Levelt, 1994; Navarrete et al., 2006; Kittredge et al., 2008; Knobel et al., 2008). In a clinical context, increased use of highly frequent words and difficulties in the production and recognition of infrequent words are found in aphasic conditions and dementia (Nickels and Howard, 1994, 1995; Bird et al., 2000; Sailor et al., 2004; Schwartz et al., 2004; Vita et al., 2014; Boukrina et al., 2015; Kavé and Goral, 2016; Faroqi-Shah and Milman, 2018).

To address this issue with respect to DBS, we sought to analyze word frequency and variations in lexical classes in spontaneous language samples obtained from two participant groups with both STN and VIM DBS while being ON and OFF stimulation as well as from healthy control persons. To this end, we planned to re-analyze the data reported by Ehlen et al. (2016). In view of the aggravated linguistic deficits associated with thalamic DBS, we hypothesized that active vs. inactive VIM DBS would produce a shift towards the production of words with higher frequency. In contrast, we did not expect STN DBS to unfold negative effects on semantic performance, in analogy to earlier findings of subtle language improvements ON as compared with OFF stimulation conditions.

## Materials and Methods

### Participants

Twenty-six participants treated with VIM or STN DBS (each *n* = 13) at the outpatient clinic for movement disorders of the neurological department of the Charité—Universitätsmedizin Berlin were included. For this study, we re-analyzed data from the VIM DBS group reported by Ehlen et al. (2016). All participants in the VIM DBS group were treated for ET; all participants with STN DBS for PD. In addition, 13 healthy individuals with no current or earlier neurological or psychiatric conditions participated in the study as a control group. General cognition in all participants was evaluated with the Parkinson Neuropsychometric Dementia Assessment (PANDA) with a maximum score of 30 points (Kalbe et al., 2008). The PANDA was also used in the VIM DBS group and healthy volunteers for reasons of comparability. A score below the cut-off value indicating cognitive impairment (18 points) was an exclusion criterion for the study. The PANDA was performed in both stimulation conditions; for the baseline comparison between groups, we used the PANDA scores obtained OFF DBS to avoid stimulation dependent effects.

For the demographic data of all participant groups see Table 1. None of the participants had any current or previous history of psychiatric or neurological disorders other than ET or PD, respectively. All participants were right-handed and native speakers in German.

**Table 1:** Sample characteristics.

	Controls	VIM DBS	STN DBS
Age (years)	67.5 ± 8.4	70.15 ± 9.2	67 ± 7.6
Age range (years)	54–78	42–79	55–77
Education (years)	10.8 ± 1.5	9.6 ± 1.7	10.15 ± 1.6
Sex (male/female)	8/5	7/3	10/3
Disease duration (years)		15.4 ± 13.6	13.7 ± 4.8
DBS duration (years)		3.5 ± 3.2	2.9 ± 1.8
PANDA (points)	27.7 ± 1.9	21.4 ± 6.3*	22.9 ± 3.6*

Note: overview of the sample characteristics (mean ± SD); significant differences (*p ≤ 0.05) between controls and DBS groups are marked with an asterisk. All participants were right-handed. The PANDA scores were obtained in the OFF condition.

All participants performed two experimental sessions, i.e., in the ON and OFF condition, in counterbalanced order with an intersession interval of 2 months. The concomitant medication (if applicable) was not significantly changed between the sessions. In the STN DBS group, all participants received dopaminergic medication with a levodopa equivalent daily dose (LEDD; Tomlinson et al., 2010) of 563 (±385) mg in the ON condition and 569 (±446) mg in the OFF condition (ON vs. OFF: *p* = 0.885). In the DBS ON condition, participants had been stable under the current DBS settings for 2 months prior to the experiment, and in the DBS OFF condition the stimulation was switched off at least 30 min before the examinations. A longer interval between switching off the stimulation and testing would have increased the strain on the participants. The healthy control group performed one experimental session.

The experiment was evaluated and reviewed by the institutional ethics committee (EA2/047/10); all participants gave their informed and written consent prior to the experiments. The research was conducted in accordance with current guidelines and the Declaration of Helsinki.

### DBS Electrode Implantation and Localization

The stereotactic surgery for the implantation of tetrapolar DBS electrodes (DBS Lead Models 3387 for VIM DBS and 3389 for STN DBS; Medtronic, Minneapolis, MN, USA) had been carried out in the department of neurosurgery of the Charité—Universitätsmedizin Berlin. After preplanning based on atlas coordinates and individual preoperative MRIs, the localization of electrodes was established using intraoperative micro-electrode recordings as well as macro-electrode stimulations and confirmed by postoperative T2w-MRIs conducted within 2 days after surgery. In one participant of the VIM DBS group, DBS electrodes had been implanted into the left hemisphere only; all remaining participants received bilateral stimulation.

The position of active electrode-contacts was determined based on the susceptibility artifacts of the DBS electrodes after normalization of post-operative MRI-data to the standardized Montreal Neurological Institute (MNI) stereotactic space. Positions within this space are defined by the medio-lateral, anterior–posterior and rostro-caudal axis relative to a central reference point. The total electrical energy delivered (TEED) was calculated using the formula (Koss et al., 2005):

$${\text{TEED}}_{1\sec}=\frac{voltage^{2}\times pulse\;width\times frequency}{impedance}\times 1\;\sec ond.$$

See Table 2 for DBS stimulation parameters and comparisons between both DBS groups.

**Table 2:** Deep Brain Stimulation (DBS) parameters.

	STN DBS		VIM DBS		STN DBS vs. VIM DBS	
	Right	Left	Right	Left	Right	Left
Amplitude (V)	2.52 ± 1.14	2.78 ± 1	3.32 ± 1.46	3.13 ± 1.48	n. s.	n. s.
Pulse width (μs)	64.62 ± 11.27	64.62 ± 11.27	70 ± 14.77	71.54 ± 15.19	n. s.	n. s.
Frequency (Hz)	119 ± 23.6	119 ± 23.6	152 ± 33.3	155 ± 33.1	*p* = 0.046	*p* = 0.026
TEED	91.5 ± 85.8	103.1 ± 75.8	220.1 ± 261.4	273.3 ± 552	n. s.	n. s.
Polarity (mono/bi)	11/2	11/2	8/4	8/5	n. s.	*p* = 0.039
Positions of active electrode contacts					
*x* (mm)	11.85 ± 0.90	−11.62 ± 0.79	14.10 ± 1.32	−13.91 ± 1.47	*p*< 0.001	*p*< 0.001
*y* (mm)	−14.39 ± 1.02	−14.53 ± 1.0	−15.46 ± 1.36	−15.47 ± 1.32	n. s.	n. s.
*z* (mm)	−7.08 ± 1.25	−6.79 ± 1.0	−1.30 ± 1.89	−1.33 ± 1.44	*p*< 0.001	*p*< 0.001

*Note: overview of the DBS parameters of both DBS groups (mean ± SD). One participant in the VIM DBS group was implanted with DBS electrodes in the left hemisphere only. The results of statistical comparisons between both DBS groups are given in the right column. TEED = total electrical energy delivered. The Electrode positions correspond to the active contacts in the Montreal Neurological Institute (MNI) stereotactic space with the medio-lateral (x), anterior–posterior (y) and rostro-caudal (z) axis in each hemisphere. n. s. = not significant*.

### Spontaneous Language Samples and Transcription

All participants performed a semi-structured interview, in the DBS groups in both ON and OFF conditions, which was digitally recorded (software: Audacity 1.3.13-beta, microphone: the t.bone MB 88U Dual). For a detailed description see also Ehlen et al. (2016; data collection in the STN DBS group and controls was identical to the VIM DBS group). In each session one out of a predefined set of six open questions for the interviews relating to: (i) school days; (ii) work; (iii) parents; (iv) home; (v) vacation and (vi) hobbies was asked in randomized order across participants and balanced across the two sessions. If a participant did not produce a monologue of at least 60 s, paused or indicated that their answers were complete, the interviewer either referred to the answer given by the participant or addressed another topic from the question set outlined above.

The interviews were transcribed for further analysis following guidelines of the “Aachener Sprachanalyse” (ASPA; Grande et al., 2006; Hussmann et al., 2012). The resulting word lists obtained from the interviews were tagged with “Part of Speech” (PoS) tags denoting their lexical class (i.e., verb forms, nouns, adjectives, etc.), according to a standard German tagset (Schiller et al., 1999). For an automated annotation of PoS-tags (Schmid, 1999) we used the software TreeTagger^1^.

### Analysis of Lexical Class and Word Frequency

To obtain a comparable number of words in the ON vs. OFF DBS states and between participants, we analyzed the first 50 words produced during the interviews, since the total number of words varied strongly and approximately 50 words were the minimal overall word count obtained in the interviews. Repetitions were excluded, so that each uttered word was included in the analysis once. Interjections, proper names (of persons or places), numbers or dates were not analyzed as their mentioning depended strongly on the subject matter of the interview or the personal background. Errors resulting in the production of non-words such as phonological paraphasias were also not included in the analysis. We calculated the mean word frequency and standard deviations for each participant and DBS ON/OFF condition.

The total number of words remaining to be analyzed was compared between DBS conditions and groups. Furthermore, we analyzed the proportion of lexical classes by differentiating open and closed class words. Open class words were defined as nouns, full verbs, adjectives, and modal adverbs; closed class words comprised modal and auxiliary verbs, all other types of adverbs, conjunctions, pronouns, particles, prepositions, and articles (Garrett, 1990; Fanselow and Staudacher, 2008; Dürscheid, 2012), for this classification see also Ehlen et al. (2016). We calculated the ratio of open to closed words for further comparisons, given that the overall number of words contained in the analysis showed no variation between stimulation conditions or groups after exclusion of repetitions and errors (see below).

For the analysis of lexical frequency, the normalized (i.e., occurrences of a given word computed per 1 million tokens within the corpus) and log_10_-transformed word frequency for each word was retrieved from the German dlexDB database^2^. The logarithmically transformed frequency was selected to obtain normally distributed data for statistical testing because word frequency data typically shows a skewed distribution (Baayen, 2012). The dlexDB database is based on the core corpus of the German reference lexicon (Digitales Wörterbuch der Deutschen Sprache^3^) and contains approximately 100 million running words (Geyken, 2007) collected from written fiction, non-fiction, scientific and newspaper texts. Compared with the widely used CELEX database it is larger, includes more modern data and provides an easy-to-use online interface (Brysbaert et al., 2011; Heister et al., 2011). We computed the mean word frequency of: (i) all words as well as separately for; (ii) open and; (iii) closed class words in each group and DBS condition.

### Statistical Analysis

All data were tested for normal distribution with the Kolmogorov–Smirnov test.

Comparisons between stimulation states in both DBS groups were conducted by means of a mixed analysis of variance (ANOVA) with DBS target (VIM/STN) as between-subjects factor and stimulation status (ON/OFF) as within-subjects factor. *Post hoc* comparisons were made by using paired or independent samples *t*-tests. In case of not normally distributed data (word ratios) non-parametric tests (i.e., Wilcoxon signed-rank or Wilcoxon rank-sum test) were used.

Group comparisons between all three participant groups, i.e., both DBS groups either ON or OFF stimulation and healthy volunteers were performed using a one-way ANOVA and, if applicable, independent-samples *t*-tests for *post hoc* comparisons. For not-normally distributed data, groups were compared on the basis of Kruskal–Wallis H and Dunn *post hoc* tests. Dichotomous data (sex, DBS polarity) was compared between groups by using the *χ*^2^-test.

In case of significant effects or interactions of the ANOVAs, we report *F*-values, degrees of freedom (*df*), *p*-value and (partial) eta squared (*η*^2^) as an estimate of the obtained effect size. All reported *p*-values of *post hoc* tests indicating a significant difference (i.e., *p* ≤ 0.05) were adjusted for multiple comparisons using the Bonferroni correction method. We used the software SPSS^TM^ version 24 (IBM) for the statistical analysis.

## Results

### Sample Characteristics

No statistically significant differences between groups regarding age, years of education or sex ratio emerged. Both DBS groups did not differ with respect to disease duration or DBS duration. There was a significant difference between controls and both DBS groups regarding the total PANDA score ($\chi_{\left(df=2\right)}^{2}$ = 14.914, *p* = 0.001). *Post hoc* test showed that total PANDA scores were lower in each DBS group when compared with controls (STN DBS—controls: *p* = 0.018; VIM DBS—controls: *p* = 0.001). Panda scores between both DBS groups did not differ significantly (*p* = 0.334).

### Analysis of Lexical Classes

**Figure 1:**
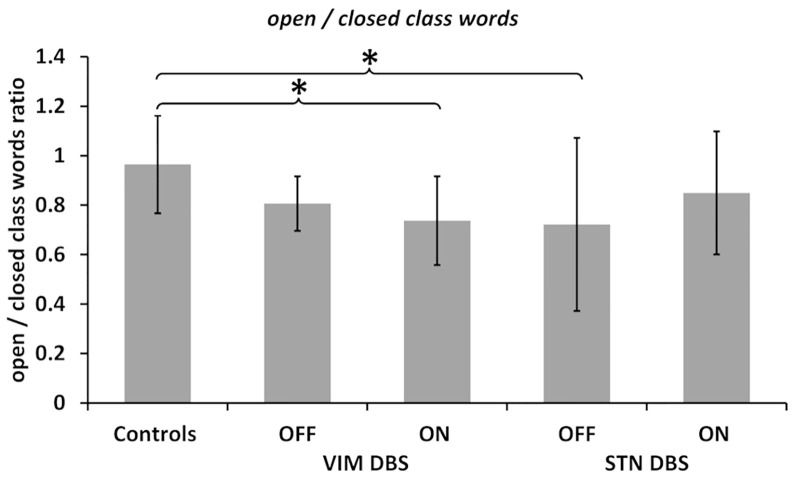
Open/closed words ratio. Ratios calculated between the number of open class words divided by the number of closed class words in all groups and deep brain stimulation (DBS) ON and OFF conditions as noted. Values are group averages with error bars indicating standard deviations. Significant (*p* < 0.05) comparisons between Controls and the DBS groups or between stimulation conditions are marked with asterisks.

In the following, we only report the results of the statistical analysis for better readability; a descriptive overview of the detailed results in each group is provided in Table 3. For a depiction of the results see Figure 1.

**Table 3:** Word frequency and lexical class.

	Cntr.	VIM DBS			STN DBS		
		OFF	ON	OFF—ON	OFF	ON	OFF—ON
Words analyzed	36.9 ± 2.2	36.5 ± 4.6	36.9 ± 2.9	n. s.	35.2 ± 4.1	36.8 ± 3.1	n. s.
Open/closed ratio	0.96 ± 0.2	0.81 ± 0.11	0.74 ± 0.18*	n. s.	0.72 ± 0.35**	0.85 ± 0.25	n. s.
Word frequency
All words	2.36 ± 0.23	2.31 ± 0.19	2.48 ± 0.17	*p* = 0.004	2.53 ± 0.2	2.39 ± 0.28	n. s.
Open class	1.44 ± 0.41	1.15 ± 0.37	1.49 ± 0.23	*p* = 0.028	1.40 ± 0.31	1.32 ± 0.48	n. s.
Closed class	3.23 ± 0.15	3.24 ± 0.23	3.19 ± 0.16	n. s.	3.28 ± 0.13	3.28 ± 0.23	n. s.

Note: results of comparisons between DBS ON and OFF conditions and across groups, i.e., testing between controls and DBS groups in both ON and OFF conditions separately. Values are the means ± SD. Significant results of *post hoc* comparisons between groups are indicated by asterisks (*p < 0.05; **p < 0.01; adjusted for multiple comparisons); see “Results” section for details. Mean word frequency is given as normalized (per 1 million tokens) and log_10_-transformed values. n. s. = not significant.

The one-way ANOVA for total number of words included in the analysis after exclusion of repetitions or unintelligible utterances did not indicate significant group-differences in both ON/OFF conditions. Likewise, the mixed ANOVA did not yield significant main effects or interactions regarding the total number of analyzed words.

The comparison of the ratio of open to closed class words between the ON and OFF condition did not reach statistical significance in either DBS group. For a comparison of potential stimulation effects between groups, we computed the difference of word ratios between the ON and OFF conditions in both DBS groups; this did not differ significantly between both groups.

The comparison across groups, on the other hand, indicated a differential effect of DBS stimulation on the ratio of open to closed class words. The Kruskal–Wallis H test yielded a significant difference between all three groups, i.e., controls and both DBS groups OFF stimulation ($\chi_{\left(2\right)}^{2}$ = 11.377, *p* = 0.003) as well as ON stimulation ($\chi_{\left(2\right)}^{2}$ = 6.888, *p* = 0.032). *Post hoc* comparisons indicated fewer open class and more closed class words (indicated by a decreased ratio) in the VIM and STN DBS OFF conditions compared to the control group. This difference was statistically significant between the STN DBS group and controls (*p* = 0.002), but did not reach significance when compared between the VIM DBS and control group (*p* = 0.101).

However, *post hoc* comparisons between controls and both DBS groups in the ON condition indicated that the VIM DBS group produced significantly fewer open class and more closed class words than controls (*p* = 0.026), whereas the difference between the STN DBS group and controls was no longer significant (*p* = 0.185); see Figure 1.

### Comparison of Lexical Frequency

**Figure 2:**
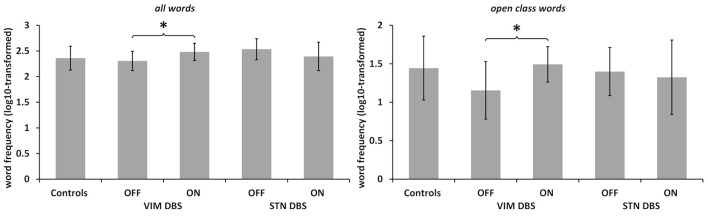
Word frequency. Log_10_-transformed word frequency in controls and both patient groups in DBS ON and OFF conditions as noted. Note the different scaling. Values are group averages with error bars indicating standard deviations. Significant (*p* < 0.05) comparisons between Controls and the DBS groups or between stimulation conditions are marked with asterisks.

See Figure 2. The mixed ANOVA run for the mean log_10_-transformed word frequency of the language samples did not yield any significant main effects, but a significant interaction of DBS target and stimulation status (*F*_(24,1)_ = 11.537, *p* = 0.002, partial *η*^2^ = 0.325). *Post hoc* comparison between ON and OFF conditions in each DBS group separately showed that in the VIM DBS groups word frequency was significantly higher in the ON than in the OFF condition (*t*_(12)_ = 3.915, *p* = 0.004). In the STN DBS group, no statistically significant difference emerged. The comparison of DBS groups during the ON condition with controls did not yield any significant differences. The one-way ANOVA conducted for the DBS OFF condition indicated a significant effect of group (*F*_(36,2)_ = 4.261; *p* = 0.025, *η*^2^ = 0.192). *Post hoc* comparisons showed a significantly lower word frequency in the VIM DBS than the STN DBS group (*p* = 0.028), but did not indicate significant differences between controls and either DBS group.

In light of the altered ratio of open to closed class words among both DBS groups, we also conducted a separate analysis of mean word frequency by lexical class between DBS conditions and groups. For open class words, the ANOVA yielded a significant interaction of DBS target and stimulation status (*F*_(24,1)_ = 4.308, *p* = 0.049, partial *η*^2^ = 0.152). *Post hoc* comparisons between ON and OFF in each DBS group separately revealed an increased lexical frequency for the VIM DBS group in the ON as compared to the OFF condition regarding open (*t*_(12)_ = 2.868, *p* = 0.028) and no significant difference in the STN DBS group. For closed class words, no significant main effects or interactions emerged. The one-way ANOVA did not indicate any significant group differences for the mean word frequency of open or closed words in both ON and OFF conditions.

## Discussion

In this study, we examined the effect of VIM and STN DBS on spontaneous language on the level of lexical frequency and use of lexical classes (i.e., open and closed class words). Our main findings are: (i) an increase of lexical frequency in the VIM DBS group under active compared to inactivated stimulation, whereas there was no significant modulation by STN DBS; (ii) a lower proportion of open class words in both DBS groups relative to controls with; (iii) opposite effects of subthalamic and thalamic DBS, that is an increase of open class words conveying semantic information by STN DBS in contrast to a (further) decrease of open class words by VIM DBS. Altogether, the pattern of results can be summarized as an indication of changed word use associated with VIM DBS and to a lesser extent also with STN DBS.

As open and closed class words differ markedly with respect to lexical frequency, the question arises how the observed increase of word frequency by active vs. inactive VIM DBS is linked to a shift towards the production of more closed class words of higher lexical frequency. The separate analysis by lexical class, however, confirmed higher word frequency of open class words by active VIM DBS but not STN DBS. Altogether, this pattern suggests at least a combination of a quantitative shift towards more closed class words and retrieval of more highly frequent words in case of thalamic DBS. Of note, in the STN DBS group there was an apparent, but not statistically significant decrease in overall word frequency in the ON vs. OFF condition (see Figure 2, Table 3). This difference was diminished when comparing the frequency of open class words between DBS conditions, which suggests that any visible, yet not significant contrast of word frequency between STN DBS conditions was, in contrast to VIM DBS, rather due to a quantitative shift of closed as compared to open class words.

Regarding the nature of open class words conveying semantic content (Garrett, 1990; Fanselow and Staudacher, 2008; Dürscheid, 2012), the modulation of word frequency by VIM DBS may reflect impaired lexico-semantic retrieval associated with thalamic stimulation. Whereas closed class words can only marginally be varied regarding their frequency, there are numerous competing alternatives for open class words during lexical selection. An increased production of more highly frequent alternatives can be taken as an index of lexical simplification of language (Paetzold and Specia, 2017). A similar shift towards high-frequency words is commonly observed in language produced by individuals suffering from dementia (Thompson-Schill et al., 1999; Bird et al., 2000; Silveri et al., 2002; Sailor et al., 2004; Forbes-McKay et al., 2005) or in speech patterns of aphasic patients (Howard et al., 1984; Nickels and Howard, 1994, 1995; Cuetos et al., 2002; Boukrina et al., 2015; Faroqi-Shah and Milman, 2018). In cases of thalamic aphasia, such substitution of words with semantically related and highly frequent alternatives has been linked with the development of semantic paraphasias and jargon (Crosson, 2019). Furthermore, although phonemic errors following thalamic lesions have been observed (Radanovic and Scaff, 2003), lexical-semantic errors appear to prevail (Nadeau and Crosson, 1997). Crosson (2019) has argued that the dominance of this error type arising at the interface between the semantic and lexical step of word selection may be due to the greater complexity with thousands of semantic concepts and words stored in the mental lexicon as opposed to a limited number of phonemes.

As to the neuroanatomical underpinnings of the observed effects, a modulation of lexical access by thalamic DBS ties in with evidence for a suggested role of medial thalamic nuclei (e.g., the centromedian-parafascicular complex) for “higher-order” cognitive and particularly language processing (Zoppelt et al., 2003; Ye et al., 2011; Liebermann et al., 2013; Llano, 2013; Pergola et al., 2013; Saalmann, 2014). Aphasic syndromes, however, have most consistently been associated with damage to the anterior as well as posterior (pulvinar) parts of the (more often left or dominant) thalamus (Karussis et al., 2000; Schmahmann, 2003; Carrera and Bogousslavsky, 2006; Fritsch et al., 2020). Finally, intraoperative electrical stimulation of the pulvinar and posterior ventrolateral regions in the left hemisphere was described to produce anomia (Hebb and Ojemann, 2013). These clinical findings are consistent with modeling of DBS current spread in patients with impaired VF output associated with VIM DBS (Ehlen et al., 2017), suggesting anterior thalamic structures rostral to the VIM nucleus as a possible locus for impaired word processing. Moreover, anterior lesions might disrupt thalamo-cortical connectivity, resulting in the disconnection of thalamic from frontal (middle frontal gyrus) and temporal regions as a potential mechanism underlying lexical deficits in patients with thalamic aphasia (Nishio et al., 2014).

From a conceptual perspective in view of the “selective engagement” theory (Nadeau and Crosson, 1997), an interpretation of the current results could be that thalamic nuclei are involved in monitoring and binding cortical activations during lexical access through cortico-thalamic-cortical circuits (Crosson, 2019). Consistent with this idea, automatic lexical activation underlying word retrieval during VF task performance has been found to be decreased by thalamic, but not subthalamic DBS (Vonberg et al., 2016; Ehlen et al., 2017). On a functional level, this would link the perturbation of thalamo-cortical networks by thalamic DBS with “spreading activation” within semantic networks thought to occur during accessing the mental lexicon (Collins and Loftus, 1975) and lemma retrieval (Roelofs, 1992).

With respect to the absence of STN DBS effects on lexical frequency, our findings seem compatible with results of earlier studies on natural language, which altogether did not indicate marked effects on lexicality either (Batens et al., 2014, 2015). Furthermore, STN DBS lead to subtle improvements of executive, but not lexical functions underlying word generation during VF tasks (Vonberg et al., 2016) as well as grammatical processing (Zanini et al., 2003, 2009); but see Phillips et al. (2012) and Schulz et al. (2012) for reports of negative effects of STN DBS on language. Consistent with most of these findings, however, linguistic abnormalities associated with basal ganglia lesions have been related to procedural dysfunctions underlying processing of grammatical or syntactic (i.e., rule-based) language properties rather than with lexico-semantic deficits (Ullman, 2004; Crosson et al., 2007; Kotz et al., 2009).

Both DBS groups differed from controls in that they showed a lower proportion of open class words. With respect to verbs, an overuse of closed class (i.e., modal and copula) verbs has been interpreted as a compensatory strategy for morphosyntactic deficits (Bastiaanse, 2011; Batens et al., 2014). Whereas the comparison between DBS groups and controls suggested a subtle, yet opposite modulation of the ratio of open to closed class words by the stimulation status, there was no significant difference between ON and OFF conditions as in earlier studies (Batens et al., 2014, 2015; Ehlen et al., 2016). This could be explained by small effect sizes and small sample sizes in the current as well as the previous investigations. The interpretation of this finding is also limited by the fact that our analysis was focused on DBS effects on lexical frequency, and that the comparison of open vs. closed class words was included for this purpose. Therefore, we did not conduct a comprehensive linguistic analysis of the whole interviews by means of calculating type-token-ratios to investigate lexical variability. As the interviews were relatively short, this question might be addressed in future studies using longer samples consisting of, for example, consecutive interviews.

Whereas a central finding of this study is a differential effect of DBS on language production in either thalamic or subthalamic stimulation-targets, it should be mentioned that the DBS groups inevitably differed with respect to the underlying pathology, being ET and PD. Thus, it should be acknowledged that either of these conditions is associated with cognitive symptoms independent from DBS (Lombardi et al., 2001; Kehagia et al., 2010; Louis, 2016; Pfeiffer, 2016). In both disorders, however, the predominant clinical phenotype of cognitive symptoms appears to be a frontal-type dysexecutive syndrome (Puertas-Martín et al., 2016; Sánchez-Ferro et al., 2017). With respect to language functions, a typical finding in both ET and PD populations is reduced word production in VF tasks (Henry and Crawford, 2004; Cersonsky et al., 2018; Ratajska et al., 2021). In spite of these similarities between ET and PD, however, the question of how cognitive symptoms of the underlying disease and dopaminergic medication (in patients with PD) might have contributed to the observed effects of either thalamic or subthalamic DBS has to remain open at this point. Of note, both DBS groups with inactive stimulation performed worse on the PANDA test assessing global cognition than controls, possibly reflecting lesioning by electrode implantation as well as the underlying pathology. However, we did not find significant differences regarding lexical frequency between controls and DBS groups as a potential factor for the current results.

With respect to relevant differences between both DBS groups in terms of stimulation settings, it is worth noting that DBS frequency was slightly higher in the VIM DBS group, which included more patients with bipolar DBS electrodes, whereas all other settings did not differ. High-frequencies (i.e., 120–150) as compared to (not therapeutically used) very low-frequencies (i.e., 10 Hz) DBS has indeed been associated with aggravated linguistic (i.e., VF) deficits (Wojtecki et al., 2006; Pedrosa et al., 2014). Having said this, DBS frequency was within the range of typical therapeutic high-frequency settings in both patient groups, and higher DBS frequency in the VIM DBS group would rather account for a gradual than the qualitative difference between both DBS groups observed here.

To conclude, the current results reveal impaired lexical selection during natural language induced by thalamic but not subthalamic DBS. This observation extends earlier findings of reduced sentence complexity and impaired lexical activation associated with VIM DBS. From a clinical perspective, increased lexical frequency under VIM DBS corresponds to similar effects observed in aphasic patients due to permanent brain lesions. Thalamic but not subthalamic DBS might mediate these effects by a perturbation of cortico-thalamo-cortical networks causing a decrease of spreading activation in lexico-semantic representations underlying or facilitating word retrieval.

## Data Availability Statement

The datasets presented in this article are not readily available because the dataset consists of recorded interviews and therefore contains potentially identifiable data; the same applies to the transcripts of these recorded interviews due to the subject matter (e.g., biography, profession). Requests to access the datasets should be directed to hannes.tiedt@charite.de.

## Ethics Statement

The studies involving human participants were reviewed and approved by Institutional ethics committee of the Charité—Universitätsmedizin Berlin (EA2/047/10). The patients/participants provided their written informed consent to participate in this study.

## Author Contributions

HOT contributed to the conception of the work, analysis and interpretation of the data, and wrote the main manuscript text. MW contributed to acquisition and analysis of the data. FE contributed to the conception of the work, acquisition, analysis and interpretation of the data, and substantively revised the article. FK contributed to the conception of the work, interpretation of the data, and substantively revised the article. All authors contributed to the article and approved the submitted version.

## Conflict of Interest

The authors declare that the research was conducted in the absence of any commercial or financial relationships that could be construed as a potential conflict of interest.

## Footnotes

^1^http://www.cis.uni-muenchen.de/~schmid/tools/TreeTagger

^2^http://www.dlexdb.de

^3^http://www.dwds.de
